# Plaque burden improves the detection of ischemic CAD over stenosis from coronary computed tomography angiography

**DOI:** 10.1007/s10554-025-03396-9

**Published:** 2025-04-22

**Authors:** Tanja Kero, Sarah Bär, Antti Saraste, Riku Klén, Jeroen J. Bax, Juhani Knuuti, Teemu Maaniitty

**Affiliations:** 1 Department of Surgical Sciences, Nuclear Medicine & PET, Uppsala University, Uppsala, Sweden; 2 Turku PET Centre, Turku University Hospital, University of Turku, Turku, Finland; 3 Department of Cardiology, Bern University Hospital Inselspital, Bern, Switzerland; 4 Heart Center, Turku University Hospital, University of Turku, Turku, Finland; 5 Department of Cardiology, Leiden University Medical Center, Leiden, The Netherlands; 6 Department of Clinical Physiology, Nuclear Medicine, and PET, Turku University Hospital, Turku, Finland; 7 PET Center / Medical Imaging Center, Uppsala University Hospital, Uppsala, 75185 Sweden

**Keywords:** Coronary CT angiography, Artificial intelligence, Coronary plaque, Ischemia, Positron emission tomography

## Abstract

In symptomatic patients undergoing coronary CTA for suspected coronary artery disease (CAD), we assessed if quantification of plaque burden, in addition to luminal narrowing and clinical risk factors, offers incremental value for the identification of ischemic CAD on a per patient level. We evaluated 2145 patients who underwent coronary CTA for suspected CAD with sequential selective downstream ^15^O-water positron emission tomography (PET) myocardial perfusion imaging. Coronary CTA scans were analyzed using Artificial Intelligence-guided Quantitative Computed Tomography (AI-QCT), with measurement of maximum diameter stenosis, percent atheroma volume (PAV), percent calcified plaque volume (CPV) and percent noncalcified plaque volume (NCPV). Ischemic CAD was defined as the presence of abnormal stress perfusion on ^15^O-water PET. PAV on top of the clinical variables and ≥ 50% stenosis improved the prediction of ischemic CAD on a per patient level as compared to clinical variables and ≥ 50% stenosis (AUC = 0.91 vs. AUC = 0.87, *p* < 0.001). The best diagnostic performance was achieved when PAV with a cut-off value of 12.2% was applied in patients with intermediate (30–70%) stenosis; using this approach, the sensitivity, specificity, positive and negative predictive values and diagnostic accuracy for ischemic CAD were 76%, 91%, 64%, 95% and 88%. The addition of quantitative plaque volume on top of clinical variables and ≥ 50% diameter stenosis improves the detection of ischemic CAD as defined by PET perfusion imaging. Applying a PAV threshold of 12.2% in patients with intermediate stenosis provided the best diagnostic performance.

**Graphical abstract:**

**Figure:**
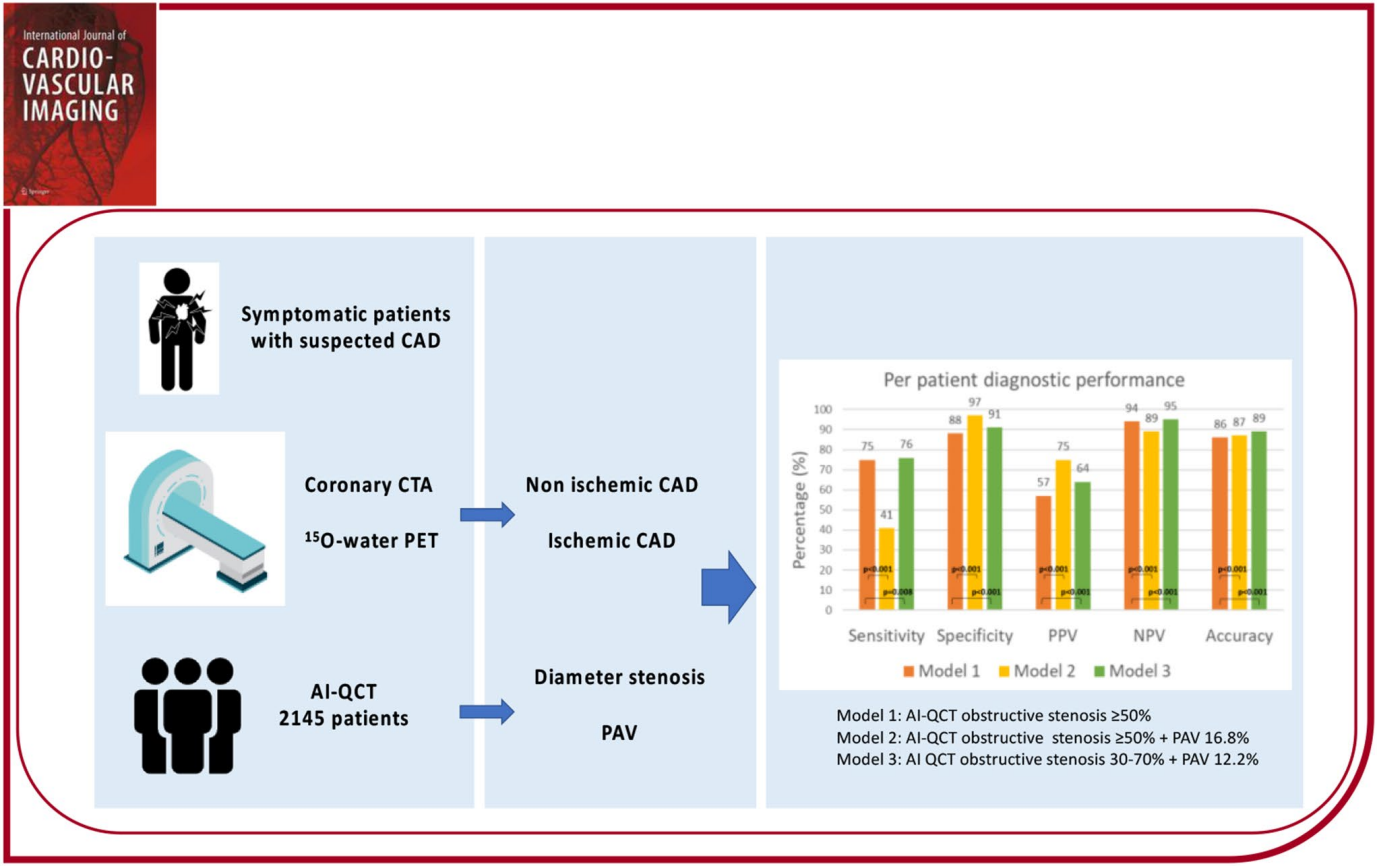
Symptomatic patients with suspected CAD underwent coronary CTA and selective ^15^O-water PET myocardial perfusion imaging. Adding AI-QCT plaque burden improved the detection of ischemic CAD in comparison with diameter stenosis only. CAD = coronary artery disease, CTA = computed tomography angiography, PET = positron emission computed tomography, AI-QCT = artificial intelligence-guided quantitative computed tomography, PAV = percent atheroma volume

**Supplementary Information:**

The online version contains supplementary material available at 10.1007/s10554-025-03396-9.

## Introduction

Coronary computed tomography angiography (CTA) has over the recent years become the first-line test for suspected coronary artery disease (CAD) in patients with a moderate or low pre-test clinical likelihood of CAD [1]. Coronary CTA has a high accuracy to rule-out obstructive CAD, but has limited specificity [2] and does not provide information on the hemodynamic consequences of CAD. Therefore, a second functional imaging modality is often used and is recommended by current guidelines to assess the presence of myocardial ischemia to guide the decision to revascularize or not [1].

Coronary CTA allows for assessment of atherosclerotic plaque quantity and morphological subtypes (calcified, non-calcified, low-density) and high-risk plaque characteristics that are known to have prognostic information [3]. Emerging evidence also suggests a relationship between plaque features and ischemia in patients with CAD [4–6]. Recently, application of artificial intelligence (AI) to the analysis of coronary CTA has enabled rapid, objective and reproducible quantification of stenosis, plaque volume and plaque characteristics with good diagnostic accuracy [7–9]. Artificial intelligence-guided quantitative computed tomography (AI-QCT) is a novel, FDA-cleared, AI-based stenosis and plaque characterization and quantification tool [7, 8]. Our hypothesis was that the measures of plaque burden with this novel tool improves prediction of ischemic CAD from coronary CTA compared with standard analysis of luminal narrowing.

Therefore, we wanted to assess if AI-QCT quantitative measures of plaque characteristics can improve the accuracy of CTA for the detection of ischemic CAD in symptomatic patients.

## Methods

### Patient population

The study population was derived from the Turku cardiac CTA registry at the Turku University Hospital, Finland. The registry includes symptomatic patients who underwent coronary CTA for suspected CAD from February 2007 to December 2016. Patients with previously known obstructive CAD or prior myocardial revascularization were not considered for inclusion. According to the institutional imaging protocol, patients with suspected CAD first undergo coronary CTA. Downstream PET myocardial perfusion imaging is performed to assess myocardial ischemia if obstructive stenosis is suspected based on the initial visual interpretation of the coronary CTA images, using ≥ 50% diameter stenosis as a guideline. For patients without visual obstructive stenosis, additional imaging is not required. The study population consists of consecutive patients, that is, both those without visual obstruction on coronary CTA (without downstream stress ^15^O-water PET) and those with suspected obstructive coronary artery stenosis on CTA (with downstream stress ^15^O-water PET). Demographic data, cardiovascular risk factors and symptoms were retrospectively collected from the medical records of Turku University Hospital.

The study complies with the Declaration of Helsinki. The Ethics Committee of the Hospital District of Southwest Finland approved the study protocol and waived the need for written informed consent.

### Hybrid coronary CTA and positron emission tomography imaging

Coronary CTA scans were performed with a 64-row hybrid PET-CT scanner (GE Discovery VCT or GE D690, General Electric Medical Systems, Waukesha, USA) as previously described [11, 12]. Before coronary CTA image acquisition, intravenous metoprolol (0–30 mg) to achieve a target heart rate of 60 bpm and oral/sublingual nitrate were administered. Coronary CTA was performed using intravenously administered low-osmolal iodine contrast agent. Prospectively triggered acquisition was applied whenever feasible. The coronary CTA scan was performed with collimation of 64 × 0.625 mm and the gantry rotation time 350 ms in all scanners. The tube current was 600–750 mA and voltage 100–120 kV according to patient size. Iterative CCTA reconstruction with standard kernel recommended by vendor was applied. Based on the initial visual evaluation of the coronary CTA findings, patients with suspected obstructive stenosis on CTA underwent dynamic quantitative PET perfusion scan during adenosine stress using a hybrid PET-CT scanner [11, 12]. Coronary CTA and PET perfusion scans were usually performed in the same session. However, some patients (8% of all) underwent PET during the following days or weeks due to logistic reasons or caffeine use (median 25 days between the scans, range 192 days). ^15^O-labeled water was used as a radiotracer and adenosine infusion (140 µg/kg/min) was used for vasodilator stress. The patients were instructed to abstain from caffeine for 24 h before the PET study.

The initial analysis of CCTA images was done by using GE ADW workstation with multiplanar reformatting and was based on visual assessment of luminal narrowing to justify the need to perform perfusion imaging. In clinical reporting PET/CT fusion images were created when necessary. The PET data were quantitatively analyzed using Carimas software (developed at Turku PET Centre, Turku, Finland) in standardized 17 segments according to American Heart Association recommendations [10, 11]. Hyperaemic MBF, expressed in mL min^− 1^g^− 1^ of perfusable myocardial tissue, was calculated for 17 myocardial segments. Myocardial stress perfusion was considered abnormal if hyperaemic MBF was ≤ 2.30 mL min^− 1^g^− 1^ in at least two adjacent segments, excluding the basal septum [12].

Ischemic CAD was defined as the presence of abnormal stress perfusion on ^15^O water PET. Absence of ischemic CAD was defined as either normal stress PET perfusion or exclusion of obstructive stenosis on clinical reading of coronary CTA.

### AI-QCT analysis

Coronary CTA scans were re-analyzed in 2022–2023 in a blinded manner using a previously described AI-based quantitative computed tomography (AI-QCT) algorithm (Cleerly LABS, Cleerly Inc., Denver, CO, USA) [7, 8]. The AI-QCT analysis was conducted in a blind manner independent of the previous visual CCTA assessment or PET perfusion findings.

This commercially available FDA-cleared software utilizes a series of validated convolutional neural networks (3D U-Net and VGG network variants) for image quality assessment, coronary artery segmentation and labelling, lumen wall evaluation and vessel contour determination, and plaque characterization.

The AI-QCT allows for assessing coronary artery lesions (areas where plaque is present). Utilizing a normal proximal reference vessel cross-sectional slice, the start and the end of the lesion, and the cross-sectional slice that demonstrates the greatest absolute narrowing, % diameter stenosis severity is automatically calculated. The most severe stenosis per-patient was selected for the current analysis. Within coronary artery lesions, plaque is quantified in a similar manner, and further characterized as low-attenuation non-calcified plaque, non-calcified plaque and calcified plaque based upon Hounsfield unit (HU) densities < 30, -189 to 350, > 350, respectively [7]. Plaque volumes (mm^3^) were calculated for each coronary lesion and then summed to compute the total plaque volume at the patient level. These plaque volumes are normalized to the individual vessel volume to obtain, referred to as plaque burden, percent atheroma volume (PAV) (%) and its components percent calcified plaque volume (CPV) (%) and percent noncalcified plaque volume (NCPV) (%). Low-attenuation plaque volumes were pooled together with non-calcified plaque volumes into NCPV (%) for the analyses in this study.

### Statistical methods

Continuous variables are shown as mean ± SD or median [interquartile range (25th–75th percentile)]. Categorical variables are shown as numbers with percentages. Mann–Whitney U test was used to compare continuous variables and two-sided Chi-square test was used for categorical variables.

Logistic regression was used to determine variables associated with myocardial ischemia. The explanatory clinical variables were chosen based on clinical reasoning and consisted of age, sex, hypertension, smoking (previous and current), dyslipidaemia, diabetes mellitus, family history of CAD and the presence of typical angina. Variables with a significant association in univariable models were included into the multivariable models. Three different measures of plaque burden were separately tested in models including plaque volumes as continuous variables: NCPV, CPV and PAV. The performances were compared based on the area under the receiver operating characteristic curves (AUC) and the plaque burden type with best performance was used in further analysis.

In order to identify (binary) cut-off values for diameter stenosis and plaque burden, diagnostic accuracy was assessed for combinations of stenosis degree and plaque burden to optimize the benefit; test characteristics for prediction of ischemia were compared for (a) Model 1: AI-QCT obstructive stenosis ≥ 50%, (b) Model 2: AI-QCT obstructive stenosis ≥ 50% + plaque volumes (%) and c) Model 3: optimized AI-QCT obstructive stenosis range + plaque volumes (%). In Model 1, the classification was based on a commonly used binary cut-off of 50% diameter stenosis (i.e., a reference model without plaque volume). In Model 2, diameter stenosis < 50% was considered as a negative finding, whereas in the presence of ≥ 50% stenosis the classification was based on plaque burden cut-offs. In Model 3, plaque burden was used for classification within the optimized (intermediate) stenosis range, whereas stenosis below this range was considered as negative and stenosis above this range was considered as a positive finding (irrespective of the plaque burden). To obtain the optimal stenosis range and threshold for plaque volumes (%), we performed a grid search. The lower and upper bounds of the stenosis range were given values from 1 to 99% with increments of 1%. The plaque volume threshold varied from 0.1 to 49.0% with increments of 0.1%. The optimal threshold values for plaque volumes (%) were selected with Youden index (J), maximizing the sum of sensitivity and specificity [13].

Analyses were two-tailed and a P-value of < 0.05 was considered statistically significant. All analyses were performed in Stata version 15 (StataCorp. 2017. Stata Statistical Software: Release 15. College Station, TX, USA: StataCorp LLC) and R version 3.6.0 with the packages haven 2.4.1 and caret_6.0–86.

## Results

### Patient population

**Fig. 1:**
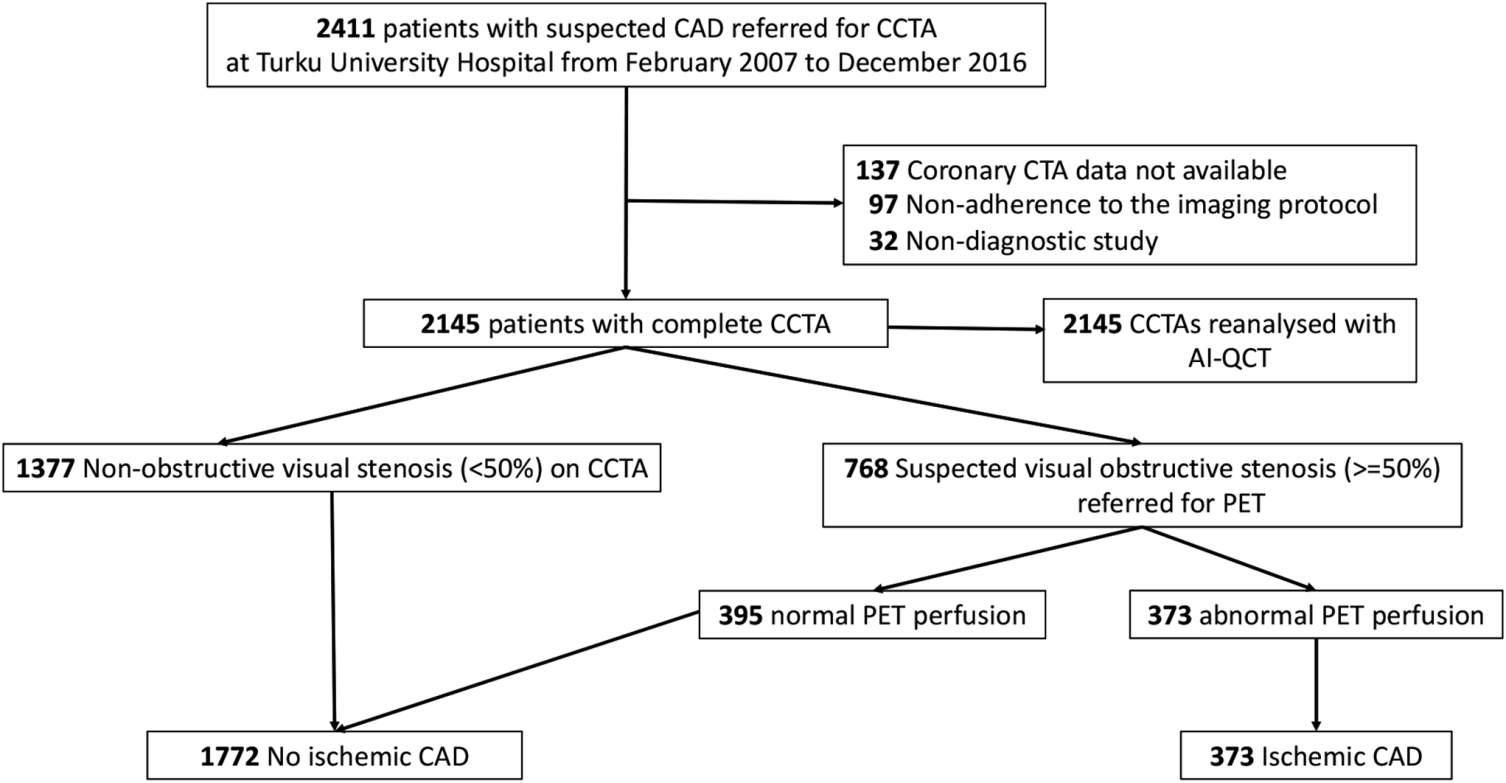
Patient flowchart on the selective hybrid CTA/PET protocol, used as a reference standard in the current study. CAD = coronary artery disease, C CTA = coronary computed tomography angiography, AI-QCT = artificial intelligence-guided quantitative computed tomography, PET = positron emission tomography

Out of the 2411 patients in the Turku CTA database, 2145 patients (89%) had coronary CTA data available for AI-QCT analysis with complete quantitative results on stenosis and plaque characteristics. The patient flowchart is shown in Fig. 1. Of the 2145 patients, 373 (17%) had ischemic CAD based on the presence of abnormal stress perfusion on ^15^O water PET. In contrast, 1772 (83%) did not have ischemic CAD, either based on the exclusion of obstructive stenosis on clinical reading of coronary CTA or based on normal stress PET perfusion.

When re-analysing the CCTA images with AI-QCT, 486/2145 patients (23%) had obstructive CAD (diameter stenosis ≥ 50%) whereas 1659/2145 patients (77%) did not have obstructive CAD (diameter stenosis < 50%). The clinical characteristics and AI-QCT findings of the patients are shown in Table 1.

**Table 1:** Patient characteristics

Patient characteristics	Ischemic CAD (*N* = 373)	No ischemic CAD (*N* = 1772)	*p*-value
Age, years	65 [59–70]	62 [56–69]	< 0.001
Sex (male), n (%)	252 (67.6%)	641 (36.2%)	< 0.001
Hypertension, n (%)	253 (67.8%)	949 (53.6%)	< 0.001
Dyslipidemia, n (%)	274 (73.5%)	1097 (61.9%)	< 0.001
Current smoker, n (%)	57 (15.3%)	206 (11.6%)	0.050
Previous smoker, n (%)	103 (27.6%)	335 (18.9%)	< 0.001
Diabetes mellitus, n (%)	81 (21.7%)	238 (13.4%)	< 0.001
Family history of CAD, n (%)	173 (46.4%)	850 (48.0%)	0.577
Typical angina, n (%)	125 (33.5%)	361 (20.4%)	< 0.001
BMI, kg/m^2^	27.7 [24.8–30.8]	27.5 [24.6–31.2]	0.981
Agatston coronary calcium score	528 [197–1243]	9 [0–108]	< 0.001
**AI-QCT**			
Diameter stenosis, %	64 [48–75]	16 [7–31]	< 0.001
Area stenosis, %	88 [74–94]	29 [12–52]	< 0.001
Total plaque volume, mm^3^	476 [264–817]	63 [22–178]	< 0.001
Non-calcified plaque volume, mm^3^	276 [170–444]	51 [20–121]	< 0.001
Low-attenuation plaque volume, mm^3^	0.1 [0.0-1.2]	0.0 [0.0–0.0]	< 0.001
Calcified plaque volume, mm^3^	158 [64–360]	5 [0–46]	< 0.001
Percent atheroma volume, %	15.7 [8.9–25.2]	2.1 [0.8–5.4]	< 0.001
Percent non-calcified plaque volume, %	8.8 [5.7–13.1]	1.7 [0.7–3.8]	< 0.001
Percent low-attenuation plaque volume, %	0.0 [0-0.04]	0 [0–0]	< 0.001
Percent calcified plaque volume, %	5.3 [0-11.4]	0.2 [0-1.5]	< 0.001
**Medication**			
Antiplatelet drug, n (%)	213 (57.1%)	725 (40.9%)	< 0.001
Lipid-lowering drug, n (%)	207 (55.5%)	653 (36.9%)	< 0.001
Betablocker, n (%)	205 (55.0%)	743 (41.9%)	< 0.001
Long-acting nitrate, n (%)	48 (12.9%)	116 (6.5%)	< 0.001
Calcium channel blocker, n (%)	76 (20.4%)	249 (14.1%)	0.002
ACE inhibitor, n (%)	86 (23.1%)	283 (16.0%)	0.001
AT II antagonist, n (%)	98 (26.3%)	337 (19.0%)	0.002

Values are n (%) or mean (± standard deviation) or median [interquartile range]. P-values are from Mann Whitney U tests or Chi-square tests. CAD = coronary artery disease, BMI = Body mass index, AI-QCT = artificial intelligence-guided quantitative computed tomography, ACE = angiotensin converting enzyme, AT II = angiotensin II

### Clinical variables and AI-QCT parameters to predict ischemia

First we looked at the performance of multivariable models in prediction of ischemia (including the clinical variables) to find out if adding plaque burden as a continuous variable would increase the performance. Age, sex, hypertension, smoking, dyslipidaemia, diabetes mellitus and typical angina were all significantly associated with ischemia in univariable regressions and were used as adjusting covariates in the multivariable regression analyses (Table 2a–c).

**Table 2:** Univariable and multivariable logistic regressions for ischemic CAD

2a. Univariable analysis			
Variable	OR	95% CI	*p*-value
Age	1.03	1.02 – 1.05	<0.001
Sex	3.67	2.90 – 4.66	<0.001
Hypertension	1.83	1.44 – 2.32	<0.001
Smoking	1.71	1.36 – 2.15	<0.001
Dyslipidemia	1.70	1.33 – 2.18	<0.001
Diabetes	1.79	1.35 – 2.37	<0.001
Typical angina	1.97	1.54 – 2.51	<0.001
Family history of CAD	0.92	0.75 – 1.17	0.577

2b. Multivariable analysis			
Variable	OR	95% CI	*p*-value
Age	1.01	0.99 – 1.03	0.291
Sex	2.69	2.01 – 3.60	<0.001
Hypertension	1.25	0.93 – 1.69	0.147
Smoking	1.26	0.94 – 1.69	0.119
Dyslipidemia	1.48	1.09 – 2.02	0.012
Diabetes	1.05	0.73 – 1.52	0.777
Typical angina	1.75	1.28 – 2.40	<0.001
Stenosis ≥50%	16.88	12.58 – 22.65	<0.001

2c. Multivariable analysis			
Variable	OR	95% CI	*p*-value
Age	0.98	0.97 – 1.00	0.086
Sex	2.07	1.51 – 2.83	<0.001
Hypertension	1.10	0.79 – 1.00	0.580
Smoking	1.00	0.73 – 1.37	0.986
Dyslipidemia	1.36	0.98 – 1.88	0.068
Diabetes	0.79	0.53 – 1.18	0.259
Typical angina	1.80	1.30 – 2.50	<0.001
Stenosis ≥50%	5.99	4.24 – 8.46	<0.001
PAV (per 1%)	1.12	1.10 – 1.15	<0.001

OR = Odds ratio, CI = confidence interval, PAV = percent atheroma volume

An obstructive AI-QCT result (diameter stenosis ≥ 50%) was significantly associated with ischemia even when adjusted for all clinical variables in a multivariable analysis (odds ratio (OR) 16.88, 95% CI 12.58–22.65, *p* < 0.001, AUC = 0.87).

PAV and NCPV on top of clinical variables had equal discriminating ability for ischemia (AUC 0.89 and AUC 0.90; p-value for between-model comparison = 0.51) whereas CPV on top of clinical variables (AUC 0.77) had significantly less discriminative ability than PAV (*p* < 0.001 for between-model comparison). Consequently, PAV was chosen for the next analyses.

PAV was significantly associated with ischemia even when adjusted for clinical variables and ≥ 50% stenosis degree in a multivariable analysis (OR 1.12 per 1% increase (CI 1.10–1.15, *p* < 0.001). Adding PAV on top of clinical variables and ≥ 50% stenosis statistically significantly improved the prediction of ischemia (AUC = 0.91 vs. AUC = 0.87, *p* < 0.001).

### Assessment of cut-off values for AI-QCT obstructive stenosis and PAV

As a next steep we identified (binary) cut-off values for diameter stenosis and PAV to make these parameters clinically useful. At this step, clinical variables were not included in the models. The ≥ 50% stenosis has been the most commonly clinically used criterion for obstructive stenosis and was used as cut-off to define positive finding in Model 1.

According to the method of Youden, the optimal PAV threshold to predict ischemia in patients with stenosis ≥ 50% was ≥ 16.8% (Model 2).

Statistically optimal stenosis range + PAV threshold according to grid search and best Youden´s J was stenosis 31–63% + PAV 7.7% (sensitivity 85%, specificity 85%, diagnostic accuracy 85%) (Online Resource, Supplemental Fig. 1a–g).

Based on these grid search results, intermediate stenosis 30–70% (simplified for clinical utility) was used as the optimized stenosis range for Model 3. In patients with intermediate stenosis 30–70% the optimal PAV threshold was ≥ 12.2%.

### Diagnostic accuracy of AI-QCT obstructive stenosis and percent atheroma volume

**Fig. 2:**
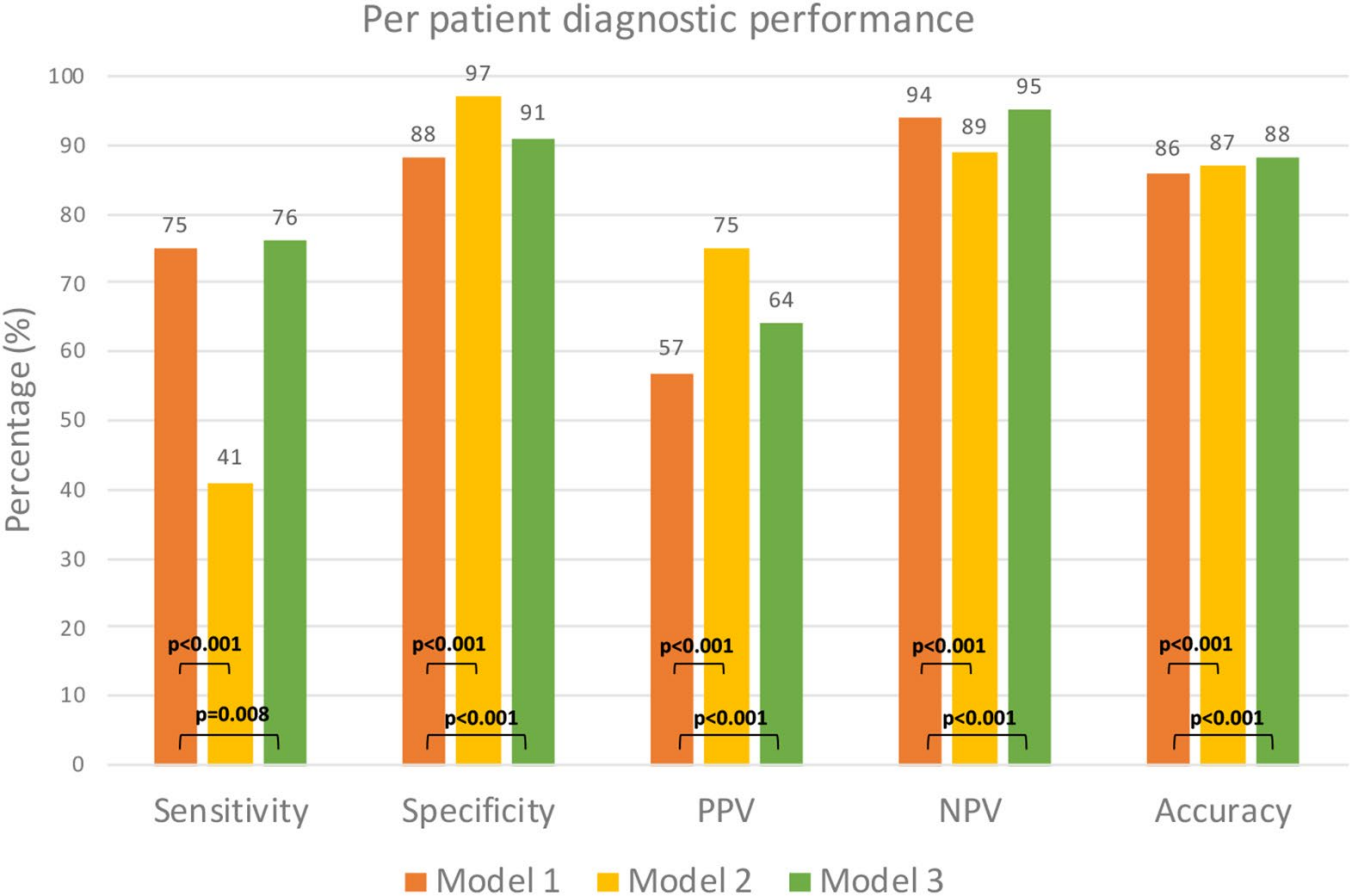
The sensitivity, specificity, positive and negative predictive values, and diagnostic accuracy of tested models. Model 1: AI-QCT obstructive stenosis ≥ 50% and Model 2: AI-QCT obstructive stenosis ≥ 50% + PAV. Model 3: AI QCT obstructive stenosis 30–70% +PAV. AI-QCT = artificial intelligence-guided quantitative computed tomography, PAV = percent atheroma volume

**Fig. 3:**
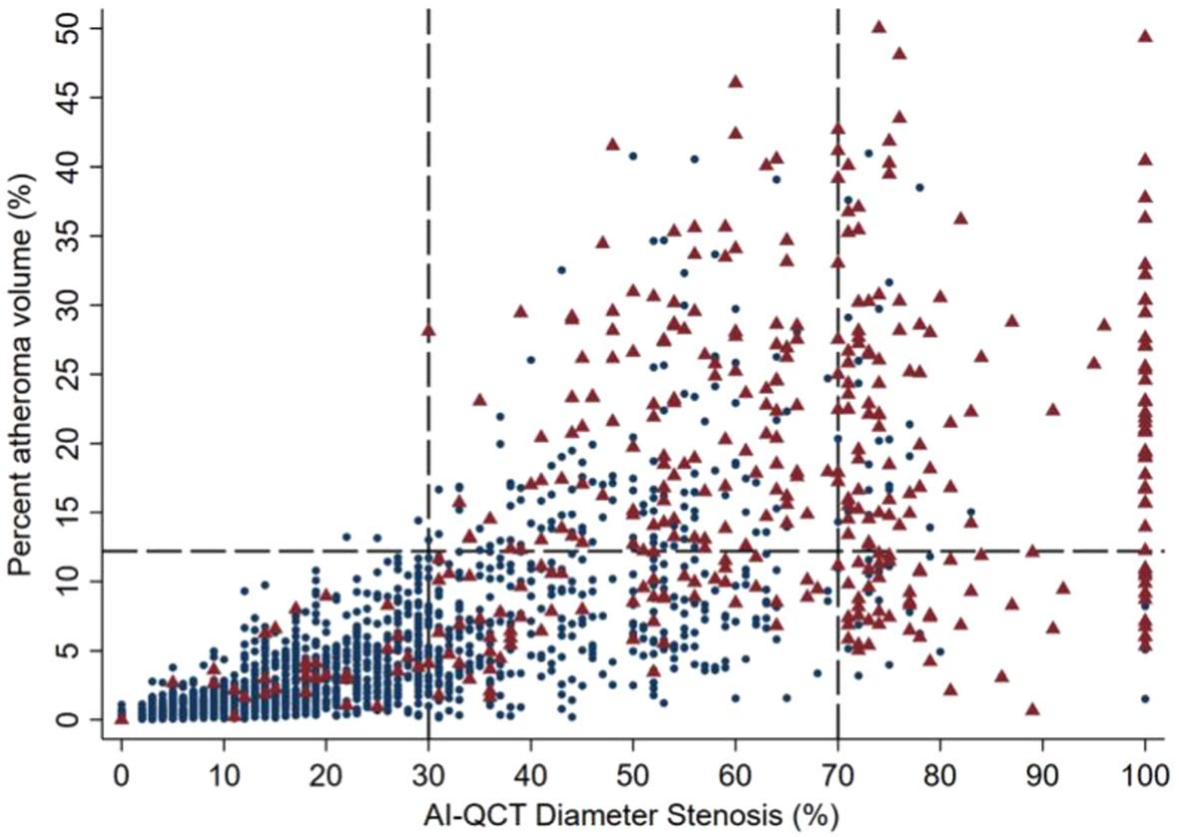
Scatterplot of AI-QCT diameter stenosis degree and PAV in patients with and without ischemia. Blue dots represent patients without ischemia as defined by PET and red triangles represent patients with ischemia. AI-QCT = artificial intelligence-guided quantitative computed tomography, PAV = percent atheroma volume, PET = positron emission tomography

Table 3; Fig. 2 shows the diagnostic accuracy of Models 1–3 and Table 4a–c shows the patient classification by each Model against ischemic CAD. Figure 3 shows the scatterplot of the degree of stenosis and PAV in patients with and without ischemia.

**Table 3:** Diagnostic accuracy for models 1–3

	Model 1 AI-QCT ≥ 50% stenosis (Reference) % (95% CI)	Model 2 AI-QCT ≥ 50% stenosis +PAV 16.8% % (95% CI)	*p*-value Model 2 vs. Model 1	Model 3 30–70% stenosis +PAV 12.2% % (95% CI)	*p*-value Model 3 vs. Model 1
Sensitivity	75 (70–79)	41 (36–42)	< 0.001	76 (72–81)	0.008
Specificity	88 (87–90)	97 (96–98)	< 0.001	91 (89–92)	< 0.001
PPV	57 (53–62)	75 (68–80)	< 0.001	64 (59–68)	< 0.001
NPV	94 (93–95)	89 (87–90)	< 0.001	95 (94–96)	0.018
Diagnostic accuracy	86 (84–87)	87 (86–89)	< 0.001	88 (87–90)	< 0.001
AUC	0.81 (0.79–0.84)	0.69 (0.67–0.72)	< 0.001	0.84 (0.81–0.86)	0.048

In Model 1, the patients were classified negative by stenosis < 50% and positive by stenosis ≥ 50%. In Model 2, the patients were classified negative by stenosis < 50% or stenosis ≥ 50% with PAV < 16.8%, and positive with stenosis ≥ 50% and PAV ≥ 16.8%. In Model 3, the patients were classified negative with stenosis < 30% and positive with stenosis > 70%. Patients with 30–70% stenosis were classified positive if PAV exceeded a binary optimal threshold (12.2%)

**Table 4:** Patient frequencies for Model 1, Model 2 and Model 3

4a: Patient frequencies for Model 1			
AI-QCT ≥ 50% stenosis	Ischemic CAD	No Ischemic CAD	Total
Positive	278	208	486
Negative	95	1564	1659
Total	373	1772	2145

4b: Patient frequencies for Model 2			
AI-QCT ≥ 50% stenosis +PAV 16.8%	Ischemic CAD	No Ischemic CAD	Total
Positive	153	52	205
Negative	220	1720	1940
Total	373	1772	2145

4c: Patient frequencies for Model 3			
AI-QCT 30-70% stenosis +PAV 12.2%	Ischemic CAD	No Ischemic CAD	Total
Positive	285	164	449
Negative	88	1608	1696
Total	373	1772	2145

In Model 1 the patients were classified negative by stenosis <50% and positive by stenosis ≥50%. In Model 2, the patients were classified negative by stenosis <50% or stenosis ≥50% with PAV <16.8%, and positive with stenosis ≥50% and PAV ≥16.8%. In Model 3, the patients were classified negative with stenosis <30% and positive with stenosis >70%. Patients with 30-70% stenosis were classified positive if PAV exceeded a binary optimal threshold (12.2%)

Model 1 (diameter stenosis ≥ 50%) detected ischemic CAD with a sensitivity, specificity, positive and negative predictive values and diagnostic accuracy of 75%, 88%, 57%, 94% and 86%, respectively.

In Model 2, the patients were classified negative by stenosis < 50% or stenosis ≥ 50% with PAV < 16.8%, and positive with stenosis ≥ 50% and PAV ≥ 16.8%. Model 2 had a higher specificity (97% vs. 88%, *p* < 0.001) and higher positive predictive value (75% vs. 57%, *p* < 0.001) in comparison to Model 1, but lower sensitivity (41% vs. 75%, *p* < 0.001) and lower negative predictive value (89% vs. 94%, *p* < 0.001). Although the diagnostic accuracy was slightly higher (87% vs. 86%, *p* < 0.001) overall, Model 2 performed worse than Model 1 in terms of area under the ROC curve (AUC = 0.69 vs. 0.81, *p* < 0.001).

In Model 3, the patients were classified negative with stenosis < 30% and positive with stenosis > 70%. Patients with 30–70% stenosis were classified positive if PAV exceeded a binary optimal threshold (12.2%). Model 3 had higher sensitivity, specificity, positive and negative predictive values, and diagnostic accuracy for ischemic CAD than Model 1 (p-value vs. Model 1): 76% (*p* = 0.008), 91% (< 0.001), 64% (< 0.001), 95% (< 0.001) and 88% (*p* < 0.001) and overall the best performance (AUC = 0.84, *p* = 0.048).

## Discussion

In this clinical cohort study, we assessed whether plaque burden and type as determined by AI-QCT, in addition to luminal narrowing and clinical risk factors offered incremental value for the identification of ischemic CAD, as defined by hybrid CTA and ^15^O-water perfusion PET.

Coronary CTA has over the recent years become the first-line test for suspected CAD when the clinical probability of the disease is moderate or low. Coronary CTA has high rule-out power for obstructive CAD, but lacks specificity. Emerging evidence suggests a relationship between plaque features and ischemia in patients with CAD [4–6]. However, the clinical use of these features has been limited due to lacking evidence of appropriate application.

The main finding of this study was that addition of PAV and NCPV to clinical variables and stenosis improved the detection of ischemic CAD. As PAV and NCPV performed similarly and as PAV has proven to be a robust quantitative variable of atherosclerotic plaque burden [14], we decided to use PAV in the further analyzes, also to improve generalizability of the results.

A vessel stenosis of ≥ 50% on coronary CTA is often used as a cut-off value for suspected obstructive CAD to select patients who may benefit from subsequent ischemia testing. In the current study a ≥ 50% stenosis yielded a sensitivity of 75%, a specificity of 88% and a diagnostic accuracy of 86% for ischemic CAD using ^15^O-water PET as a reference standard. Adding PAV (AI-QCT ≥ 50% stenosis + PAV 16.8%) increased the specificity to 97% but the sensitivity dropped to 41% and the diagnostic accuracy did not change significantly in comparison to using ≥ 50% stenosis only to predict ischemia.

Consequently, we performed a comprehensive grid search to find the optimal range of stenosis, in which adding PAV information improved the accuracy for the detection of ischemia. We found that a statistically optimal obstructive stenosis range of 31–63% together with PAV 7.7% had a sensitivity of 85%, specificity of 85% and diagnostic accuracy of 85%. Apparently, for a clinical analysis a stenosis range of 31–63% is not practical. Therefore, in the clinical decision-making, a visually less than 30% stenosis could be justified to rule out and over 70% stenosis to rule in obstructive CAD. Of note, the stenosis severity of 30–70% has been commonly considered as intermediate warranting further functional testing (either non-invasive or invasive) [15].

Based on findings in the current study, we recommend to use a PAV cut-off value of 12.2% in the patients with a stenosis ranging between 30% and 70% for the prediction of obstructive CAD. The diagnostic accuracy (88%) of this approach was the best, and better than for the ≥ 50% stenosis + PAV model. The specificity of this approach was also good (91%), and the sensitivity remained reasonably high (76%). As compared to a 50% stenosis approach the increase in sensitivity however was small with an increase of true positive cases from 278 to 285. The main clinical value is the increased specificity with a decrease of false positive cases (164 instead of 208); thus in this clinical cohort 44 patients could have avoided further functional testing. The incremental value of PAV can be also easily seen in Fig. 3a scatterplot showing the relationship of stenosis degree and PAV in patients with and without ischemia. The visual impression is that PAV improves diagnostic performance in patients with an intermediate stenosis, whereas the yield of PAV seems to be low in patients with low-grade and high-grade stenosis.

Application of artificial intelligence to the analysis of coronary CTA has enabled rapid, objective and reproducible quantification of luminal stenosis and plaque morphology and burden. Advanced machine learning methods using combinations of numerous quantitative measures from coronary CTA images has been also developed to predict ischemia [5, 6, 16]. Recently the diagnostic accuracy of a new AI-QCT_ischemia_ algorithm was tested against invasive FFR [17] in two different patient cohorts and the per-patient level sensitivity was 85–92%, specificity 56–72% and accuracy 72–81%. These results are similar to our diagnostic findings (sensitivity 76%, specificity 91%, and accuracy 88%). Of note, our findings are based on more simple and likely more robust analysis of stenosis degree and total atheroma volume only.

In the current study, we focused on diagnostic value of plaque features at the patient level. The obvious goal was to study if the plaque features improve the detection of ischemic CAD, and thereby help in the clinical decision-making for further testing or therapeutic decisions. The severity of ischemia and the impact of plaque features on clinical outcomes warrant future research.

### Limitations

This was a single-center, observational study with the limitations of a retrospective analysis. These limitations are partly compensated for by a fairly large sample size and re-analysis of coronary artery CTA scans by AI-QCT blinded to clinical data and PET perfusion results. Coronary CTA was performed during 2007–2016 on a 64 slice CT scanner. There have been technical advances in the scanners improving the spatial and temporal resolution. The results of this study were derived and tested in the same patient cohort, however the dataset was not divided into a derivation and test-cohort and the results need to be validated in an external cohort.

In our study, 36% of patients underwent downstream PET myocardial perfusion scan due to suspected obstructive coronary stenosis on CTA, a decision which was based on a prompt visual evaluation of coronary CTA images using ≥ 50% diameter stenosis as a guideline. However, blinded AI-QCT analysis revealed obstructive ≥ 50% diameter stenosis in only 23% of patients. It is possible that stenosis degree has been overestimated by the prompt visual CTA analysis, that is a known limitation of CTA [2]. However, this would result in performing some unnecessary PET perfusion scans rather than missing truly obstructive CAD causing myocardial ischemia.

The results in this study are based on a patient-level analysis as it can be considered the most robust analysis. In addition, to be able to use detailed plaque assessment e.g. in planning further treatment interventions, further analysis on a vessel-level is also warranted. Finally, it would be interesting to study whether the main epicardial vessels differ in respect to association of plaque characteristics and ischemia.

## Conclusions

The addition of AI-QCT derived PAV to clinical variables and coronary artery stenosis improves the detection of ischemic CAD as defined by PET perfusion imaging. Applying a PAV threshold of 12.2% in patients with intermediate stenosis (30–70%) was found to provide best performance and improved the diagnostic accuracy as compared to the traditional ≥ 50% stenosis approach.

## Electronic supplementary material

Below is the link to the electronic supplementary material.

Supplementary Material 1

## Data Availability

No datasets were generated or analysed during the current study.

## Abbreviations

CAD: Coronary artery disease
CTA: Computed tomography angiography
PET: Positron emission tomography
AI-QCT: Artificial Intelligence-guided Quantitative Computed Tomography
PAV: Percent atheroma volume
CPV: Percent calcified plaque volume
NCPV: Percent noncalcified plaque volume
LAPV: Percent low-attenuation plaque volume
MBF: Myocardial blood flow
FFR: Fractional flow reserve
