# Supplementary material for: Plaque burden improves the detection of ischemic CAD over stenosis from coronary computed tomography angiography
Source: Int J Cardiovasc Imaging. 2025 Apr 22;41(6):1131–40. doi: 10.1007/s10554-025-03396-9 (PMC12162699; doi:10.1007/s10554-025-03396-9)
Supplement: Supplementary file 1 — Supplementary Material 1 [file 10554_2025_3396_MOESM1_ESM.pdf]

Plaque burden improves the detection of ischemic CAD over stenosis from coronary computed tomography angiography

The International Journal of Cardiovascular Imaging

Tanja Kero[1]*, Sarah Bär[2,3]*, Antti Saraste[2,4], Riku Klén[2], Jeroen J. Bax[5], Juhani Knuuti[2,6], Teemu Maaniitty[2,6]

[1]Nuclear Medicine & PET, Department of Surgical Sciences, Uppsala University, Uppsala, Sweden;
[2]Turku PET Centre, Turku University Hospital, University of Turku, Turku, Finland;
[3]Department of Cardiology, Bern University Hospital Inselspital, Bern, Switzerland;
[4]Heart Center, Turku University Hospital, University of Turku, Turku, Finland.
[5]Department of Cardiology, Leiden University Medical Center, Leiden, The Netherlands;
[6]Department of Clinical Physiology, Nuclear Medicine, and PET, Turku University Hospital, Turku, Finland;

*The authors contributed equally to this work.

Address for correspondence:
Dr Tanja Kero
E-mail: tanja.kero@uu.se

Supplemental Figure 1 a-g. Results from grid search showing the sensitivity and specificity of different PAV thresholds, stenosis range upper bounds and stenosis range lower bounds.

PAV = percent atheroma volume.

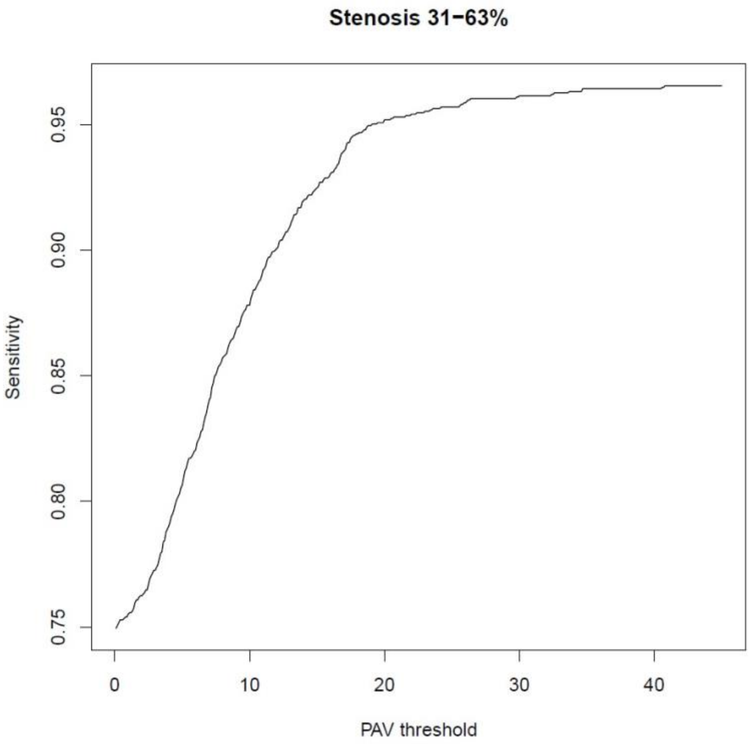

Supplemental Figure 1a.

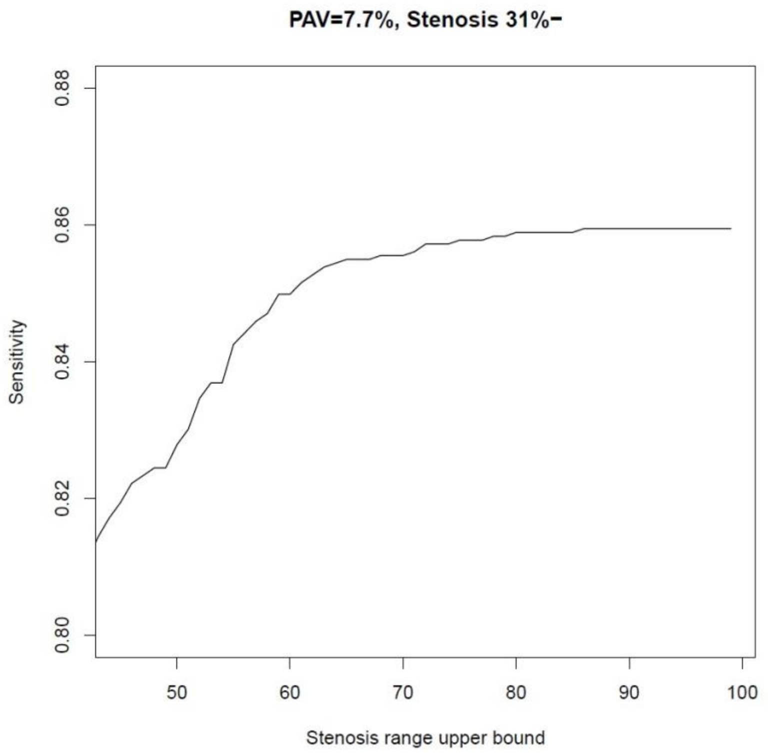

Supplemental Figure 1b.

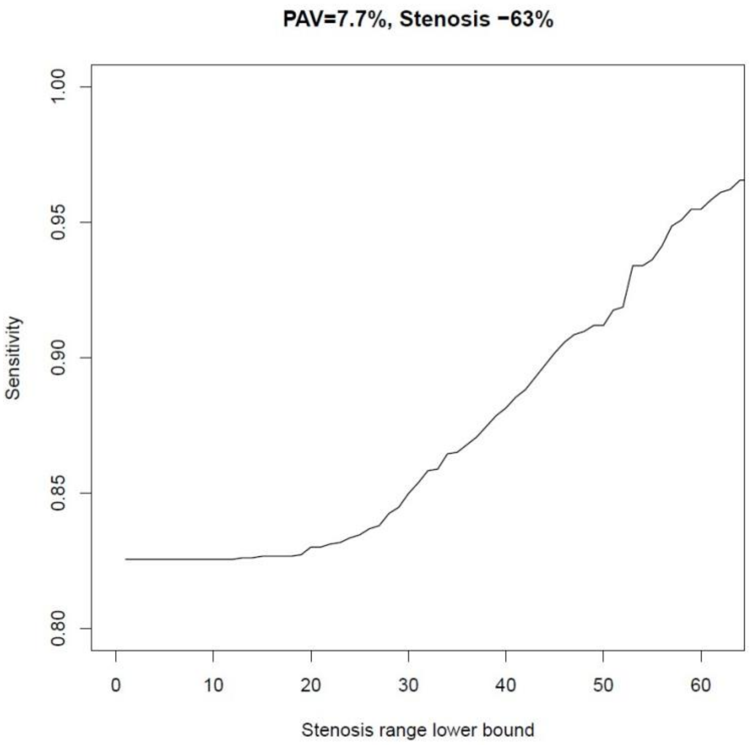

Supplemental Figure 1c.

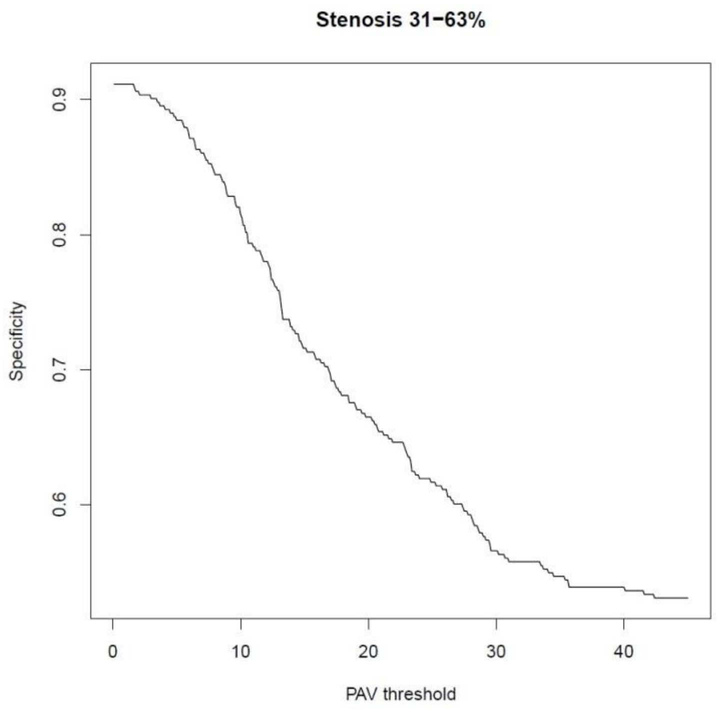

Supplemental Figure 1d.

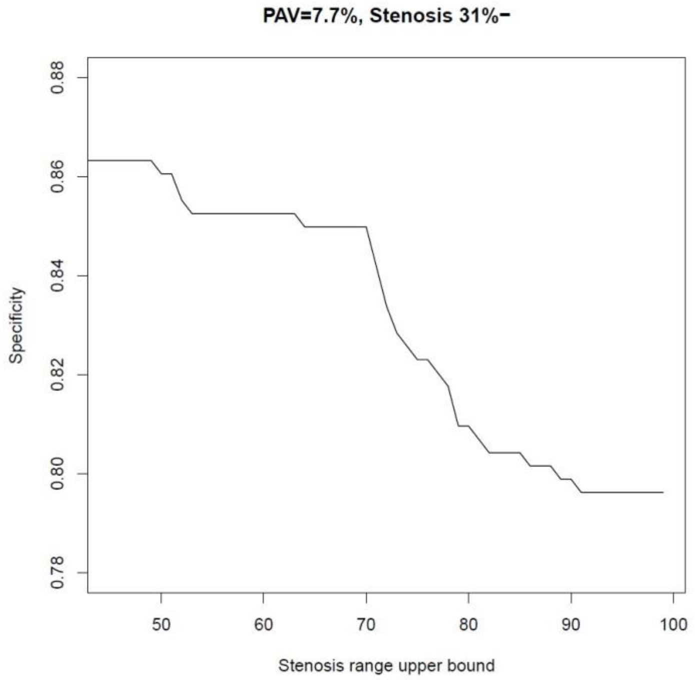

Supplemental Figure 1e.

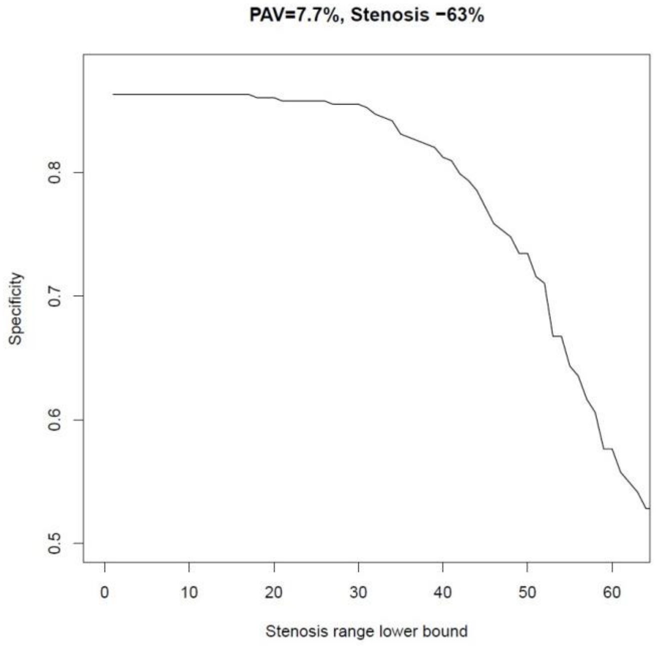

Supplemental Figure 1f.

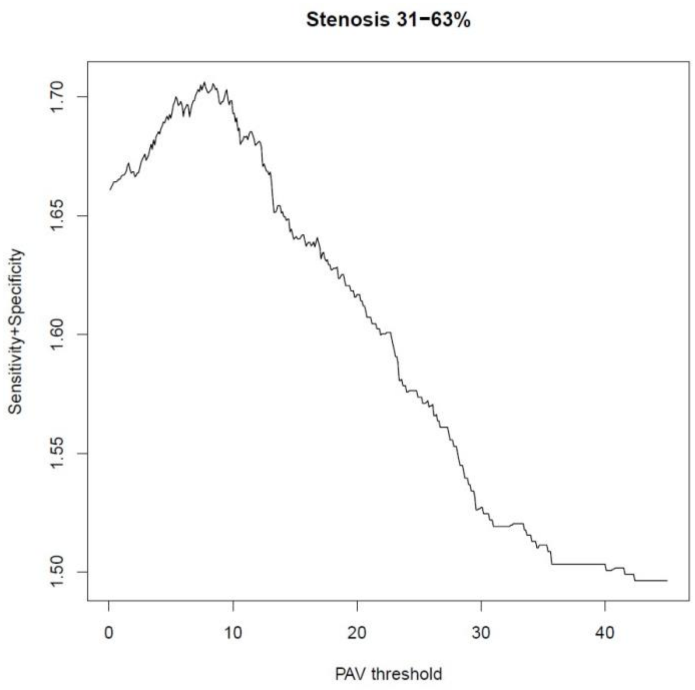

Supplemental Figure 1g.
